# Development and assessment of an RNA editing-based risk model for the prognosis of cervical cancer patients

**DOI:** 10.1097/MD.0000000000038116

**Published:** 2024-05-10

**Authors:** Zihan Zhu, Jing Lu

**Affiliations:** a Department of Biostatistics, School of Public Health, Nanjing Medical University 101 Longmian Avenue, Nanjing, P.R. China; b Department of Gynecology, The First Affiliated Hospital of Nanjing Medical University, Nanjing, Jiangsu, China.

**Keywords:** cervical squamous cell carcinoma and endocervical adenocarcinoma, prognostic model, RNA editing, tumor immune microenvironment

## Abstract

RNA editing, as an epigenetic mechanism, exhibits a strong correlation with the occurrence and development of cancers. Nevertheless, few studies have been conducted to investigate the impact of RNA editing on cervical squamous cell carcinoma and endocervical adenocarcinoma (CESC). In order to study the connection between RNA editing and CESC patients’ prognoses, we obtained CESC-related information from The Cancer Genome Atlas (TCGA) database and randomly allocated the patients into the training group or testing group. An RNA editing-based risk model for CESC patients was established by Cox regression analysis and least absolute shrinkage and selection operator (LASSO). According to the median score generated by this RNA editing-based risk model, patients were categorized into subgroups with high and low risks. We further constructed the nomogram by risk scores and clinical characteristics and analyzed the impact of RNA editing levels on host gene expression levels and adenosine deaminase acting on RNA. Finally, we also compared the biological functions and pathways of differentially expressed genes (DEGs) between different subgroups by enrichment analysis. In this risk model, we screened out 6 RNA editing sites with significant prognostic value. The constructed nomogram performed well in forecasting patients’ prognoses. Furthermore, the level of RNA editing at the prognostic site exhibited a strong correlation with host gene expression. In the high-risk subgroup, we observed multiple biological functions and pathways associated with immune response, cell proliferation, and tumor progression. This study establishes an RNA editing-based risk model that helps forecast patients’ prognoses and offers a new understanding of the underlying mechanism of RNA editing in CESC.

## 1. Introduction

Cervical squamous cell carcinoma and endocervical adenocarcinoma (CESC) are among the most prevalent types of cancer affecting women globally, ranking fourth in both incidence and mortality.^[1]^ Squamous cell carcinoma and adenocarcinoma are the predominant histological subtypes in CESC, comprising around 70% and 25% of CESC cases, respectively.^[2,3]^ CESC develops primarily due to continued human papillomavirus (HPV) infection,^[4,5]^ and thus is often prevented through HPV screening and vaccination.^[3,6,7]^ Nevertheless, HPV testing is negative in a small number of CESC cases, which include true HPV-negative cancer patients and false-negative cases.^[8–10]^ This has some detrimental implications for strategies based on HPV screening and vaccination to prevent CESC. In clinical practice, treatment methods for CESC patients mainly involve hysterectomy, chemotherapy, radiotherapy or adjuvant therapy.^[3]^ Although patients with early-stage CESC have relatively good outcomes after treatment, those with advanced, metastatic, or recurrent CESC have poor therapeutic effects.^[11,12]^ Some studies have shown that the construction of prognostic models can be used to monitor the cancer progression of CESC patients and guide precise individualized treatment to improve the patients’ prognoses.^[13–15]^ Similarly, we can find potential and important biomarkers as prognostic indicators to better prevent the development, metastasis and recurrence of CESC.

RNA editing, which occurs as a post- or co-transcriptional modification, alters specific RNA sequences and enhances the multiformity of transcriptome and proteome.^[16]^ Besides, RNA editing is mainly divided into 2 primary forms, namely adenosine to inosine (A-to-I) and cytidine to uracil (C-to-U). Among these, A-to-I RNA editing is more prevalent and catalyzed by enzymes known as adenosine deaminases acting on RNA (ADARs).^[17,18]^ RNA editing can affect the stability, structure, and function of RNA, thereby impacting protein synthesis and regulating protein function.^[19,20]^ Therefore, due to its capacity to instruct precise modifications in protein function and its underlying mechanisms, RNA editing is considered a well-positioned tool for adapting to the environment.^[20]^

As sequencing technology continues to evolve, the significance of RNA editing in cancers has been realized by an increasing number of studies. The study has discovered that some RNA editing sites are associated with tumors, and RNA editing events affect drug sensitivity.^[21]^ Moreover, RNA editing is able to influence the proliferation, metastasis, and immune response in tumor cell, thereby further impacting both malignant tumor progression and treatment response.^[22,23]^ Compared to the matched normal tissue, there is an elevation in RNA editing activity across a majority of tumor types.^[24]^ In order to assist tumor cells in adapting to different microenvironments and disease states, RNA editing levels may dynamically alter due to microenvironmental factors or tumor progression.^[19,21,25,26]^ Some studies have indicated a correlation between the occurrence and progression of malignant tumor and RNA editing, such as in the cases of hepatocellular carcinoma, colorectal cancer, and esophageal squamous cell carcinoma.^[27–29]^ However, there are no studies on the construction of prognostic models for CESC by utilizing RNA editing as a factor with predictive value, and the role of RNA editing in the prognosis of CESC has not been fully explored.

Based on our study, prognosis-related RNA editing sites in CESC were first determined to establish and evaluate corresponding prognostic models. The nomogram used to forecast patients’ prognoses was further established by RNA editing-based risk score and clinical characteristics, and its prognostic ability was verified. Finally, the underlying mechanisms of RNA editing sites in CESC were also explored.

## 2. Materials and methods

### 2.1. Data acquisition

We obtained clinical information and RNA-seq data for CESC patients from The Cancer Genome Atlas (TCGA) database (https://portal.gdc.cancer.gov/). The corresponding RNA editing profiles of CESC patients were acquired from the synapse website (https://www.synapse.org/#!Synapse:syn4382383).^[21]^ In addition, we removed cases with incomplete clinical information and RNA editing sites with missing editing levels in over 30% of samples. After processing the dataset, the TCGA dataset was divided into a training group and a testing group according to a 1:1 ratio. The TCGA training group was adapted to establish a risk model based on RNA editing, while the TCGA testing group was used to verify the prognostic capability of model.

### 2.2. Establishment of RNA editing-based risk model

To screen for sites with potential prognostic value, univariate Cox regression analysis was performed on overall survival (OS) in the training group. The detailed chromosomal locations of these sites were presented in the Manhattan plot through the “CMplot” package. Afterwards, LASSO regression analysis was performed on the screened RNA editing sites to avoid model overfitting by the “glmnet” package.^[30]^ Furthermore, we used multivariate Cox regression analysis to select the sites with prominent prognostic value by stepwise regression method.^[31]^ According to the final multivariate Cox model, we calculated risk scores by using the following method: $risk\;score=\sum_{i=1}^{n}\left(Coe_{i}*Exp_{i}\right)$, where *Exp*_*i*_ represented the editing level of sites, and *Coe*_*i*_ represented the multivariate Cox regression coefficient of corresponding sites.

### 2.3. Validation of RNA editing-based risk model

Patients were classified to either the subgroup with high-risk or the subgroup with low-risk by comparing the individual risk scores with the calculated median risk score. Afterwards, scatter plots were applied to study the connection between survival status and RNA editing-based risk scores, and heat maps were utilized to show the editing level of prognostic sites among different risk subgroups. Kaplan–Meier analysis was employed to compare OS and progression-free survival (PFS) between different subgroups by “survminer” and “survival” packages.

### 2.4. Relationship between risk scores and various clinicopathological characteristics

In order to investigate the association between risk scores and various clinical characteristics, Student *t* test was employed to compare the differences in risk scores among different ages, tumor stages, and tumor grades. Also, we conducted univariate and multivariate Cox regression analyses on both RNA editing-based scores and clinical features to evaluate whether this score could be served as an independent predictive indicator.

### 2.5. Establishment and assessment of prognostic nomogram

We combined RNA editing-based risk scores and basic clinical features to build the nomogram by means of “rms” package,^[32]^ which can be used as the quantitative tool for forecasting patients’ prognoses in real-world clinical settings. Afterwards, the predictive performance of nomogram was assessed through various methods including calibration curves, concordance index (C-index), and receiver operating characteristic (ROC) curves. Finally, to determine whether the nomogram can be of practical value in clinical decision-making, we conducted a decision curve analysis.^[33]^

### 2.6. Correlation between RNA editing site and host gene expression

By using RNA editing data and RNA-seq data, we investigated the correlation between the level of RNA editing at the prognostic site and host gene expression. Since the ADAR family of enzymes was the key enzyme used in RNA editing,^[34]^ the association of risk scores with ADAR expression level was also investigated.

### 2.7. Enrichment analysis of differentially expressed genes (DEGs)

We employed a threshold of |log2 FC| > 1 and *P* < .05 to identify DEGs between different subgroups by using “limma” package. Afterwards, Gene ontology (GO) enrichment analysis was applied to explore the biological properties of DEGs, including the biological process (BP), cellular component (CC) and molecular function (MF). Kyoto Encyclopedia of Genes and Genomes (KEGG) enrichment analysis was also performed to find pathways associated with DEGs.^[35]^ Based on the “c2.cp.kegg.symbols.gmt” and “c5.go.symbols.gmt” gene sets in the Molecular Signatures Database, we performed a gene set enrichment analysis (GSEA) on the whole genome to study the potential mechanism by which RNA editing site may exert their effects in cervical carcinoma.^[36,37]^

### 2.8. Statistical analysis

We utilized the chi-squared test to assess whether there were differences in the proportions of patients with distinct clinical features between the training and testing groups. The relationship between the 2 factors was explored by Spearman correlation analysis. In this research, we carried out statistical analyses by making use of R software (version 4.0.5), and statistical significance was defined as *P* < .05.

## 3. Result

### 3.1. Establishment and validation of RNA editing-based risk model

**Figure 1.**
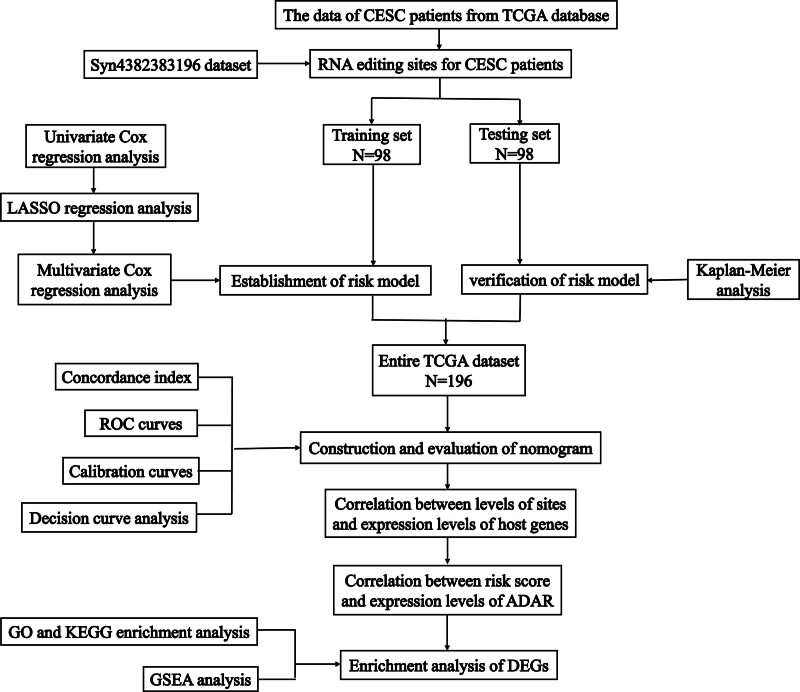
The workflow chart of this study.

The flow chart of our research is displayed below (Fig. 1). To construct and verify the prognostic model, we obtained 196 samples with RNA editing data and valid clinical information. These samples were then randomly split into the training and testing groups in equal proportion. Table 1 shows the baseline features of 2 groups. From the table, we observed no statistically significant differences in all clinical features between the training and testing groups.

**Table 1:** Baseline characteristics of patients in the training group and testing group.

Covariates	Type	Total	Test	Train	*P* value
Age	≤65	181 (92.35%)	89 (90.82%)	92 (93.88%)	.591
	>65	15 (7.65%)	9 (9.18%)	6 (6.12%)	
Grade	G1–2	103 (52.55%)	51 (52.04%)	52 (53.06%)	.6855
	G3–4	84 (42.86%)	45 (45.92%)	39 (39.8%)	
	Unknown	9 (4.59%)	2 (2.04%)	7 (7.14%)	
Stage	Stage I–II	155 (79.08%)	77 (78.57%)	78 (79.59%)	1
	Stage III–IV	37 (18.88%)	19 (19.39%)	18 (18.37%)	
	Unknown	4 (2.04%)	2 (2.04%)	2 (2.04%)	

**Figure 2.**
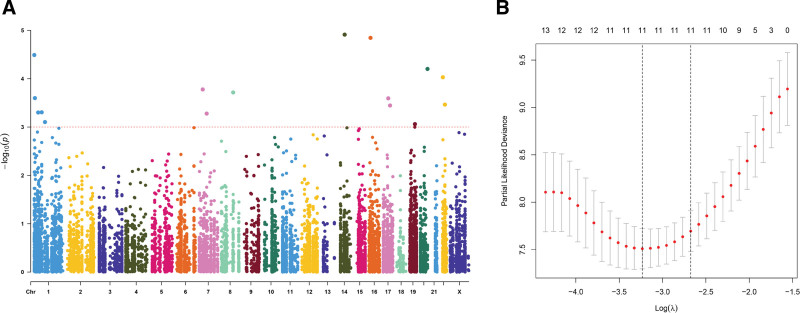
Preliminary screening of RNA editing sites related to prognosis. (A) The Manhattan plot showing the chromosomal locations of 16 RNA editing sites screened by univariate Cox regression analysis. (B) LASSO regression: the curve of Partial likelihood deviance changing with Log (λ), the smaller the value, the better the model fitting. LASSO = least absolute shrinkage and selection operator.


In order to establish an RNA editing-based risk model for forecasting CESC patients’ prognoses, we conducted univariate analysis on all RNA editing sites to initially screen the sites related to prognosis in the training group. By univariate analysis, we confirmed 16 RNA editing sites that demonstrated a strong association with patients’ OS (*P* < .001). Then, the Manhattan plot demonstrated these prognosis-related sites and their chromosomal locations (Fig. 2A). To avoid model overfitting, the above 16 RNA editing sites were moved into LASSO regression analysis, resulting in the identification of 11 sites with prognostic value based on the least partial likelihood deviation (Fig. 2B). Subsequently, 11 sites were further included in multivariate analysis, and finally 6 sites with significantly prognostic value were obtained. These 6 sites and corresponding regression coefficients were employed to build the following prognostic risk model:

$$\begin{array}{l} \begin{array}{llllll} & risk\;score=\left(11.57\times editing\;level\;of\;ICMT|chr1:6282238\right)+(35.80\times editing\; & level\;of\;APOL1\left|chr22:36662801)+(8.17\times editing\;level\;of\;PRR11\right|chr17:57279068)+\; & \left(-33.05\times editing\;level\;of\;ASB16-AS1|chr17:42259075\right)+\; & \left(15.41\times editing\;level\;of\;PPP1R13L|chr19:45896816\right)+\; & \left(29.96\times editing\;level\;of\;ATG14\;|\;chr14:55834429\right)\; \end{array} \end{array}$$

**Figure 3.**
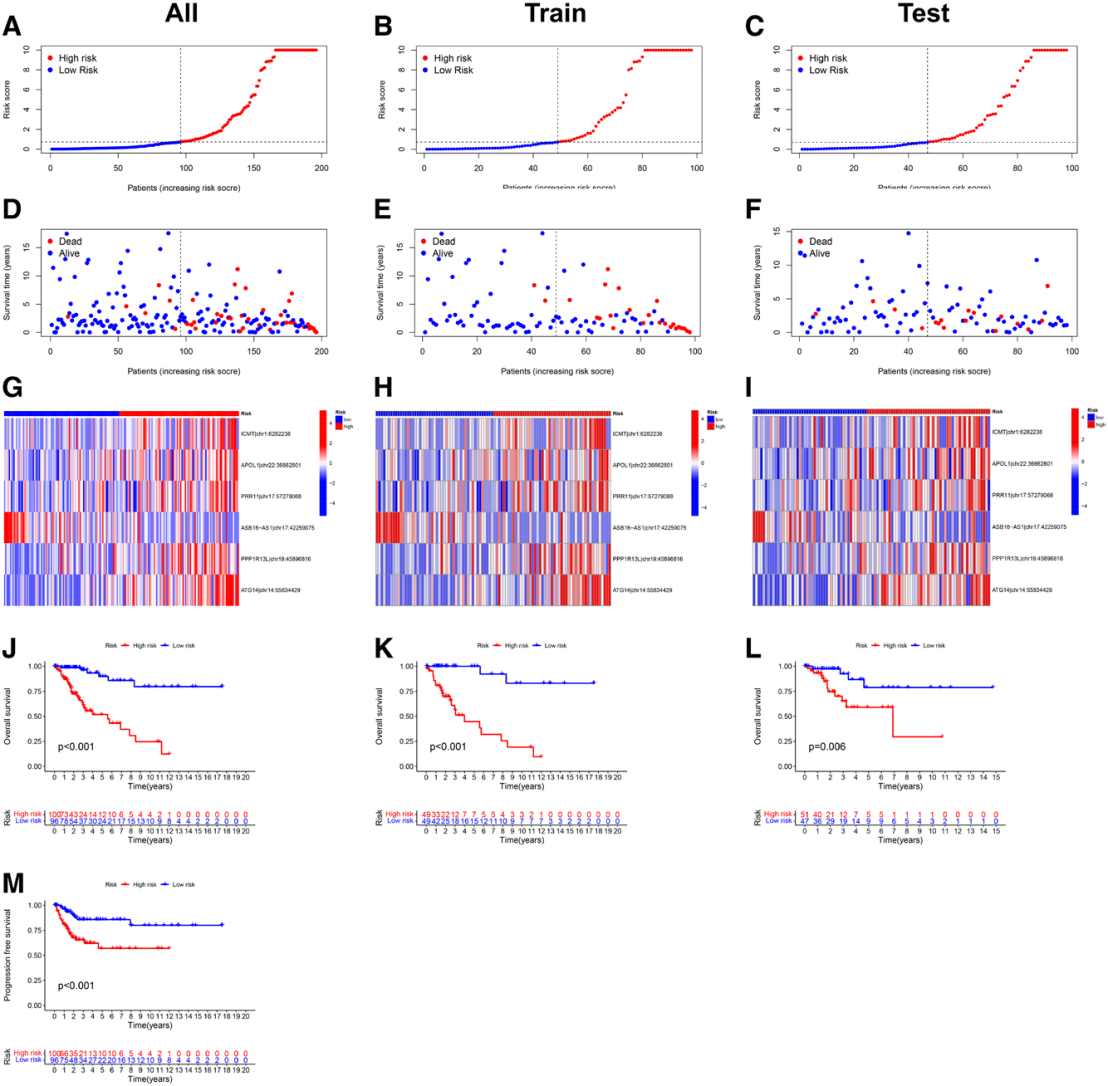
Assessing the prognostic value of the risk model in the entire dataset, training group, and testing group. (A–C) Distribution of risk score based on RNA editing prognostic model. (D-F) Survival status based on different risk scores. (G–I) Editing levels of 6 RNA editing sites in different risk groups. (J–L) Kaplan–Meier curves showing OS probabilities for different risk groups. (M) Kaplan–Meier curves showing PFS probabilities for different risk groups in the entire dataset. PFS = progression-free survival.

To test the accuracy and reliability of the risk model, the differences in survival status and editing levels of 6 prognostic sites among different risk populations were analyzed in the training group, testing group, and entire dataset. The findings indicated that low-risk patients had a longer survival time and a higher proportion of survival than high-risk patients across 3 datasets (Fig. 3A–F). The heatmap revealed that the editing level of ASB16 − AS1|chr17:42259075 generally showed a decreasing trend with increasing risk scores, while the editing levels of the other 5 sites showed the opposite trend (Fig. 3G–I). Moreover, the outcome of the survival analysis revealed that low-risk patients had a better prognosis than high-risk patients across the training group, testing group, and entire dataset (Fig. 3J–L). In the entire dataset, low-risk patients possessed a higher probability of PFS than patients with high-risk (Fig. 3M). The results above revealed that this risk model possessed good prognostic ability.

### 3.2. Relationship between risk score and different clinical features

**Figure 4.**
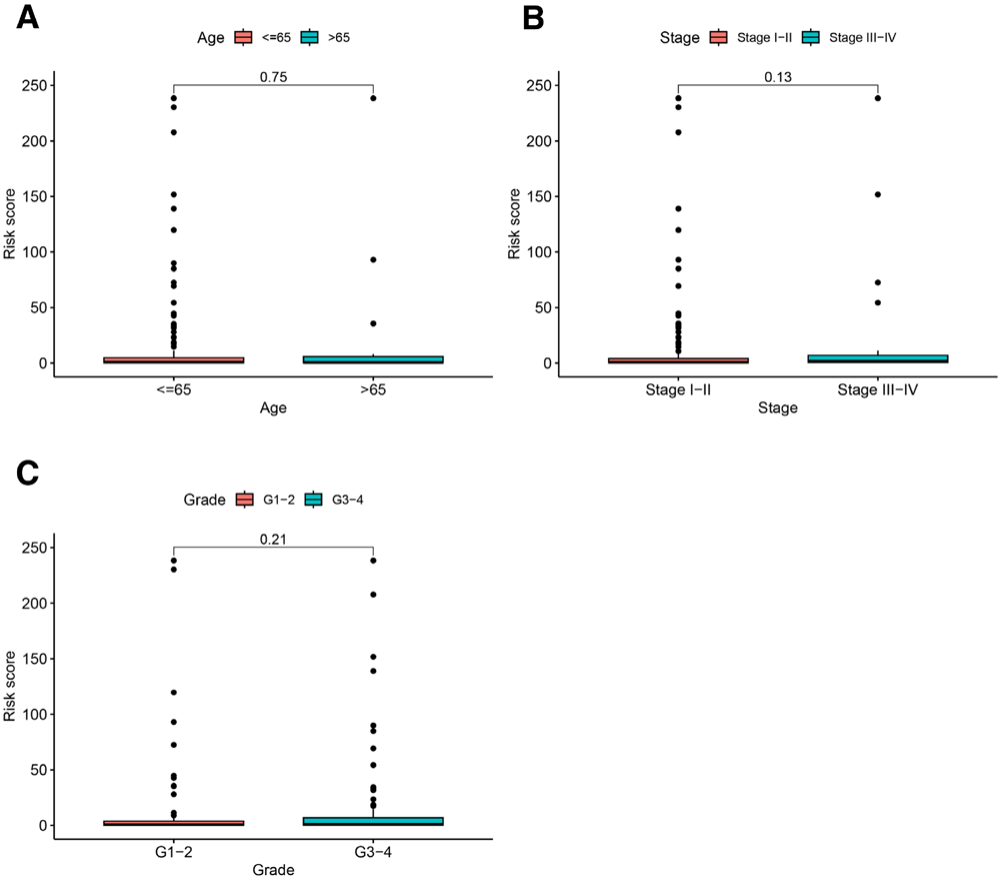
The relationship between risk score and different clinical features. (A) Analysis of differences in risk scores across age groups. (B) Analysis of differences in risk scores among different tumor stage groups. (C) Analysis of differences in risk scores between different tumor grade groups.

We analyzed the relationship between risk scores and different clinical features by using the entire dataset. The results displayed no significant differences in risk scores between different age groups, tumor stage groups, and tumor grade groups (*P* > .05, Fig. 4). We assessed the independent predictive ability of risk score and different clinical characteristics by making use of univariate and multivariate Cox analyses (Table 2). According to the outcomes derived from univariate analysis, we found that risk scores were significantly correlated with OS in the entire dataset (*P* < .001, HR = 1.009 (95%CI: 1.006 − 1.012)). The result of further multivariate analysis revealed that risk score was an independent predictor for CESC patients (*P* < .001, HR = 1.009 (95%CI: 1.006 − 1.013)), which possessed significant value in forecasting patients’ prognoses.

**Table 2:** Univariate and multivariate Cox regression analyses of the prognosis-related factors.

Variables	Univariate analysis			Multivariate analysis		
	HR	95%CI	*P* value	HR	95%CI	*P* value
Entire TCGA dataset						
Age	1.004	0.978–1.031	.756	0.988	0.961–1.016	.409
Grade	0.976	0.582–1.636	.926	0.947	0.551–1.628	.845
Stage	1.141	0.813–1.599	.446	1.087	0.754–1.566	.656
Risk score	1.009	1.006–1.012	<.001	1.009	1.006–1.013	<.001

TCGA = The Cancer Genome Atlas.

### 3.3. Construction and validation of RNA editing-based nomogram

**Figure 5.**
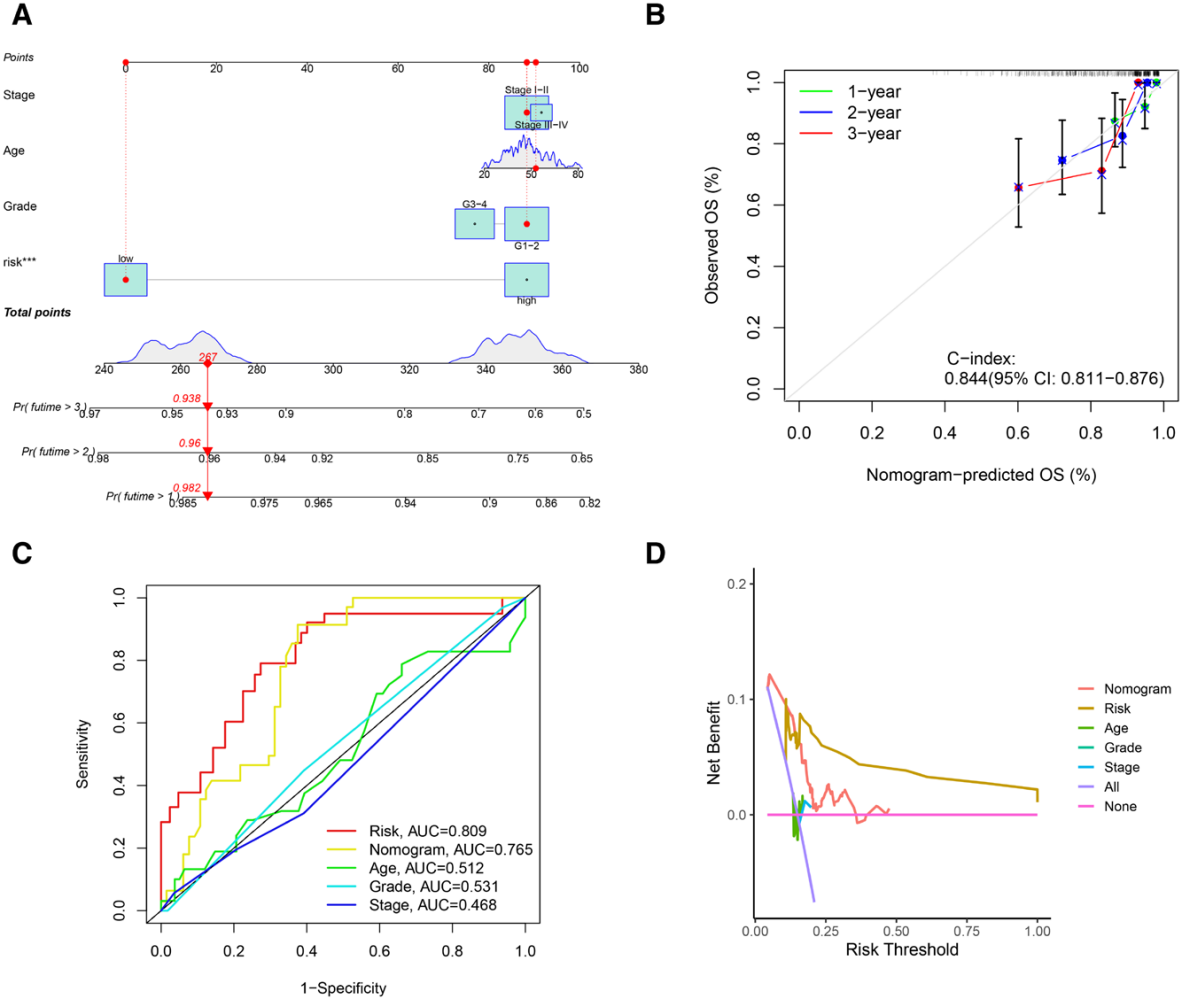
Establishment and evaluation of the nomogram based on risk score and clinical characteristics. (A) The nomogram to predict 1-, 2-, and 3-yr OS probabilities in CESC patients. (B) Calibration curves were applied to assess the 1-, 2- and 3-yr OS predictive accuracy of the nomograms in the entire dataset. (C) ROC curves showed AUC values for risk score, nomogram, age, tumor stage and tumor grade in the entire dataset. (D) Decision curves displayed the comparison of the net benefits of clinical decisions based on risk score, nomogram, age, tumor stage and tumor grade. AUC = area under the curve, CESC = cervical squamous cell carcinoma and endocervical adenocarcinoma, ROC = receiver operating characteristic.

According to RNA editing-based risk scores and clinical characteristics, the nomogram was established to improve the value of the prognostic model in clinical practice (Fig. 5A). By utilizing the nomogram, we could make predictions for 1-, 2- and 3-year survival probabilities of CESC patients. Calibration plot revealed the strong concordance between predicted and observed values (Fig. 5B). Across the entire dataset, the C-index was 0.844 (95% CI: 0.811 − 0.876), which further validated the good prognostic ability of the nomogram. Besides, ROC curves showed that risk scores (area under the curve [AUC] = 0.809) and the nomogram (AUC = 0.765) had higher area under the curve (AUC) values (Fig. 5C). This result revealed that the prognostic accuracies of risk scores and the nomogram were better than other clinical features. Finally, results of decision curve analysis indicated that the nomogram possessed relatively high clinical value (Fig. 5D).

### 3.4. Correlation between RNA editing site and host gene expression

**Figure 6.**
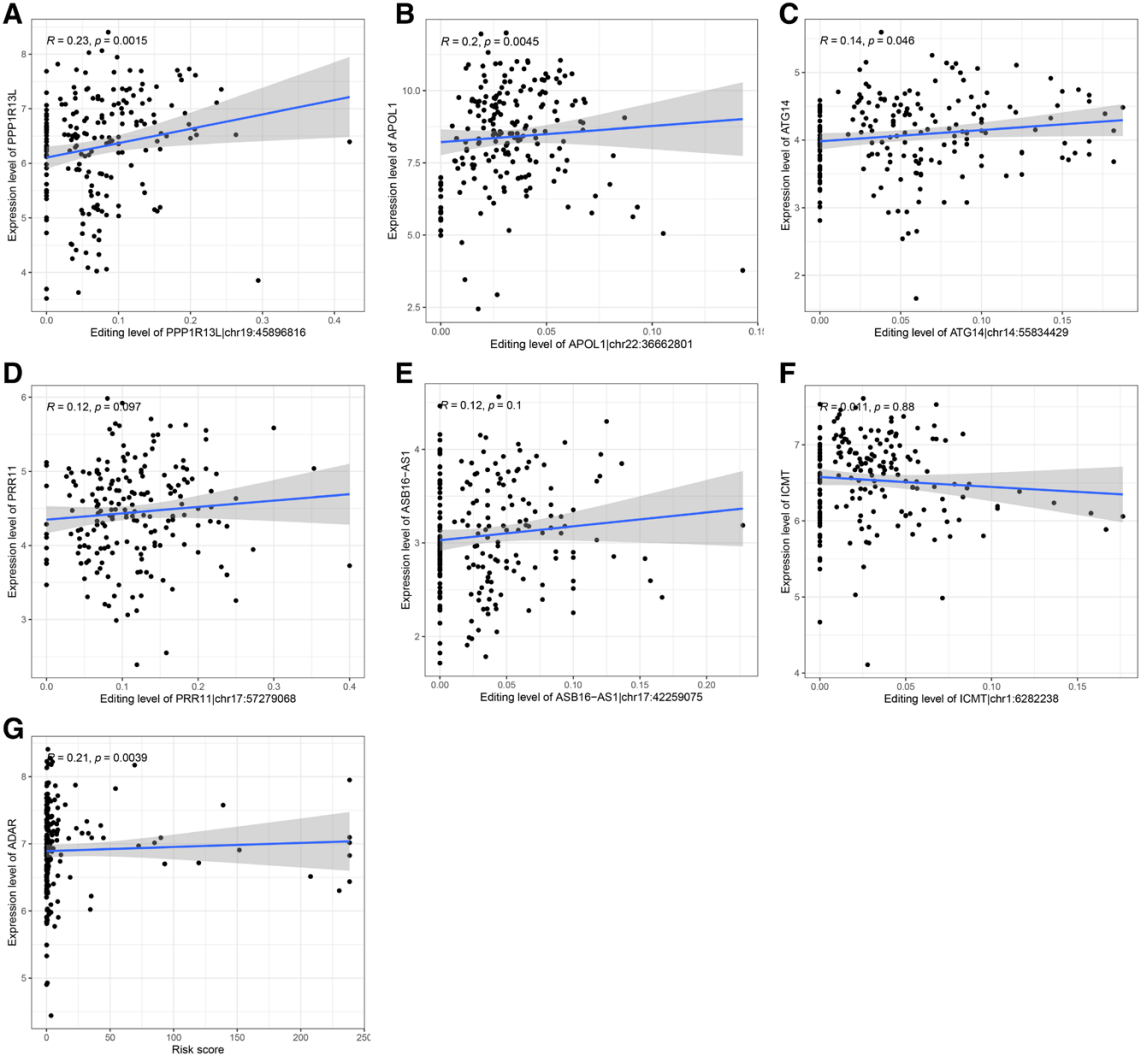
Relationship between RNA editing sites in the prognostic model and the expression of host genes. (A–F) Correlation analyses between editing levels of 6 prognostic sites and expression levels of host genes. (G) Correlation analysis of RNA editing-based risk score with ADAR expression. ADARs = adenosine deaminases acting on RNA.

The result indicated a significantly positive association between the level of RNA editing at PPP1R13L|chr19:45896816 and PPP1R13 expression level (*R* = 0.23, *P* = .0015, Fig. 6A). Also, a significantly positive association between the editing level of APOL1|chr22:36662801 and APOL1 expression level was discovered (*R* = 0.2, *P* = .0045, Fig. 6B). Figure 6C illustrated that the editing level of ATG14|chr14:55834429 was significantly positively associated with ATG14 expression level (*R* = 0.14, *P* = .046). However, the editing levels of ASB16 − AS1|chr17:42259075, ICMT|chr1:6282238, and PRR11|chr17:57279068 did not show significant correlations with their host genes (Fig. 6D–F). Furthermore, we found a significantly positive association between RNA editing-based risk score and ADAR expression level (*R* = 0.21, *P* = .0039, Fig. 6G).

### 3.5. Identification of DEGs and functional enrichment analysis

**Figure 7.**
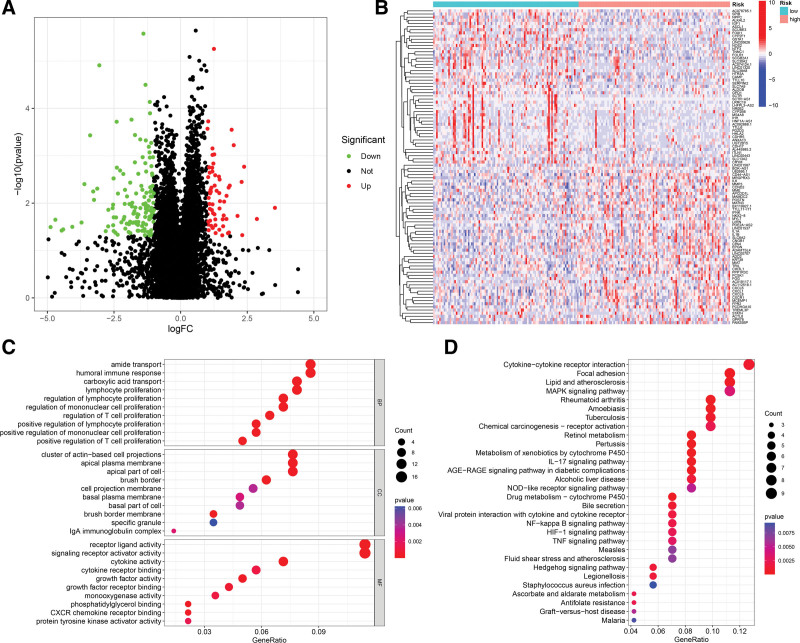
GO and KEGG enrichment analyses of DEGs among different risk groups. (A) Volcano plot revealing DEGs between high- and low-risk groups, where red and green indicated up- and down-regulated DEGs in the high-risk group, respectively. (B) Heatmap showing the expression of the top 50 up- and down-regulated DEGs in the high-risk group. (C) GO enrichment analysis of DEGs, including BP analysis, CC analysis and MF analysis. (D) KEGG analysis to assess enriched pathways of DEGs. DEGs = differentially expressed genes.

According to pre-set thresholds, we first carried out differential gene expression analysis to explore the biological functions and pathways of DEGs between different subgroups. The result indicated that 64 up-regulated and 136 down-regulated DEGs were recognized in the high-risk subgroup (Fig. 7A). Moreover, the expression of the top 50 significantly up- and down-regulated DEGs was shown by heatmap (Fig. 7B). BP analysis in GO enrichment analysis suggested that DEGs primarily participated in immune-related biological processes, including immune cell proliferation, regulation of immune cell proliferation, amide transport, and humoral immune response (Fig. 7C). The result of CC analysis revealed that DEGs were mainly enriched in cluster of actin − based cell projections, apical plasma membrane and apical part of cell (Fig. 7C). MF analysis presented that DEGs were enriched in various molecular activities and molecular receptor binding like receptor ligand activity, signaling receptor activator activity and cytokine receptor binding (Fig. 7C). Afterwards, the result of KEGG analysis displayed that DEGs were related to cytokine − cytokine receptor interaction, MAPK signaling pathway, IL − 17 signaling pathway, NF − kappa B signaling pathway, and TNF signaling pathway (Fig. 7D).

### 3.6. Gene set enrichment analysis

**Figure 8.**
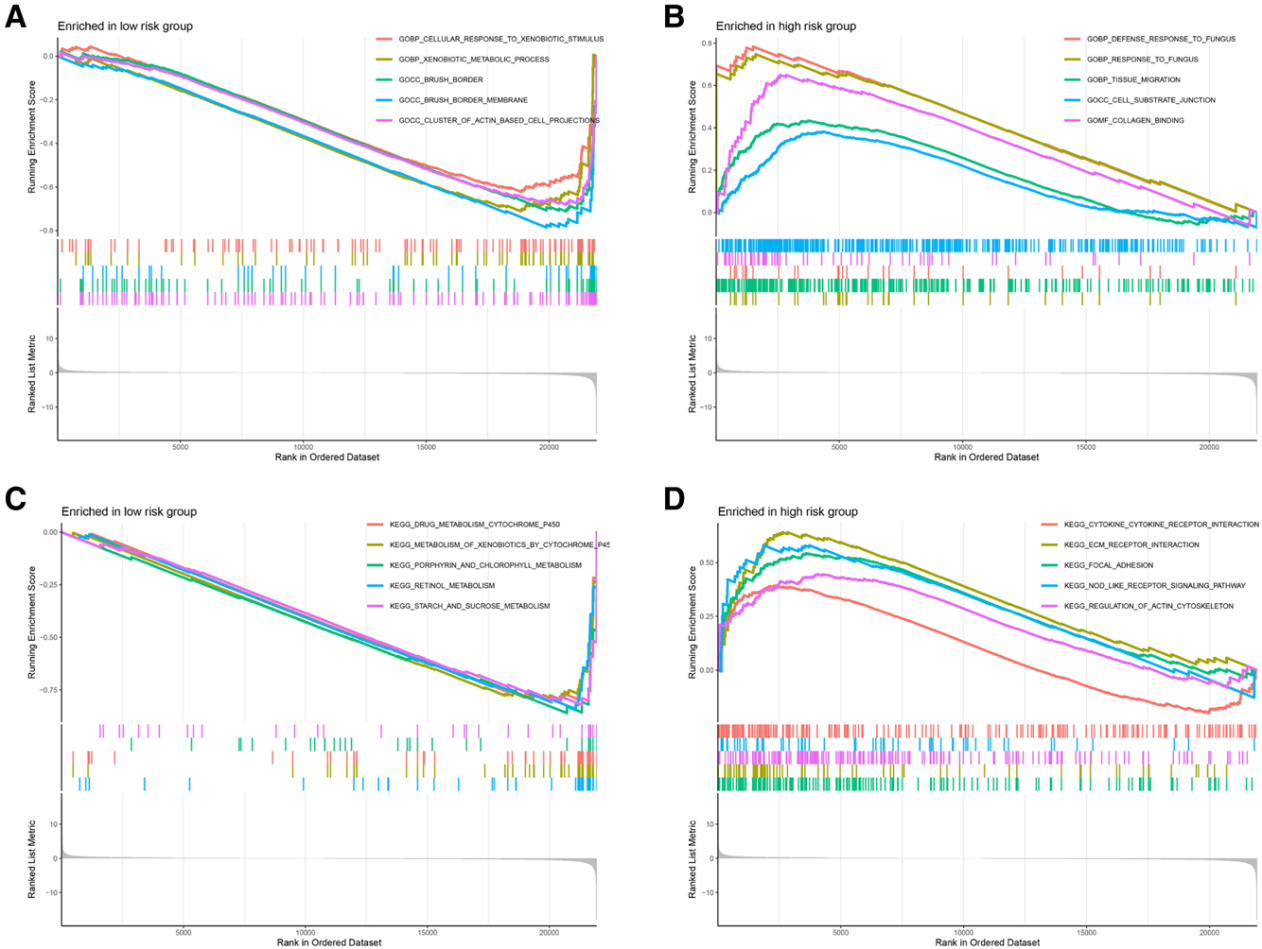
GSEA of DEGs in different risk groups. (A, B) Biological functions are significantly associated with DEGs in low-risk and high-risk groups. (C, D) Enriched pathways of DEGs in low-risk and high-risk groups. DEGs = differentially expressed genes.

To further compare the biological functions and enriched pathways of DEGs among different risk subgroups, we conducted GSEA on the whole genome. Through the analysis, we found that DEGs within the low-risk subgroup were significantly associated with biological functions such as cellular response to xenobiotic stimulus, xenobiotic metabolic process, brush border, brush border membrane and the cluster of actin-based cell projection (Fig. 8A). As shown in Figure 8B, biological functions such as response to fungus, tissue migration, cell-substrate junction and collagen binding were up-regulated in the subgroup with high-risk. Besides, DEGs in the subgroup with low-risk were mainly enriched in metabolism-related pathways (Fig. 8C). In contrast, DEGs in the high-risk subgroup were mainly enriched in cytokine-cytokine receptor interaction, ECM receptor interaction, regulation of actin cytoskeleton, focal adhesion and NOD-like receptor signaling pathway (Fig. 8D).

## 4. Discussion

Cervical cancer is a prevalent malignancy affecting the female reproductive system, with relatively high morbidity and mortality worldwide. Despite the success of the HPV prophylactic vaccine in reducing morbidity, the exact mechanisms of viral infection remain unclear. Furthermore, complex genetic and epigenetic alterations in host cell genes play a critical role in the progression of cervical precancerous lesions to invasive cancer.^[38,39]^ RNA editing, serving as an epigenetic mechanism, is closely connected to the occurrence and development of tumors.^[40]^ Also, RNA editing is one of the most prevalent genetic regulation methods in normal physiological processes. By altering the RNA sequences, it makes them different from the corresponding template genomic DNA, which influences the diversity and complexity of biomolecules.^[16,19,41]^ Because RNA editing affects RNA levels, RNA localization, alternative splicing, translation efficiency, and amino acid sequences of proteins, dysregulation of its editing process plays a key role in cancers.^[40,42,43]^ Therefore, we considered constructing a risk model through RNA editing to assess patients’ prognoses.

In the study, we finally identified 6 RNA editing sites as the best prognostic indicators of CESC through Cox and LASSO regression analyses. Then, the RNA editing-based risk model was established to stratify CESC patients with different prognoses. RNA editing can regulate physiological and pathological processes by influencing host gene expression. The relevant host genes in the risk model are all associated with cancers. APOL1 encodes a secreted high-density lipoprotein, which has been regarded as an aberrantly expressed gene in multiple cancers.^[44,45]^ Some studies found that suppression of ICMT resulted in induction of autophagy, inhibition of cell growth and inhibition of proliferation in various cancer cell types.^[46,47]^ Since PRR11 acts as an oncogenic factor in cellular proliferation, migration, invasion, cell-cycle progression, apoptosis and autophagy in some cancers, it is considered a promising prognostic biomarker.^[48]^ It has been reported that ASB16-AS1 is an oncogenic lncRNA in CESC, which affects the proliferation, migration and invasion of CESC cells by targeting miR-1305.^[49]^ Moreover, the study has shown that elevated levels of PPP1R13L can increase tumorigenesis and affect the migration of tumor cells.^[50]^ It has been reported that overexpression of ATG14 significantly enhances cell proliferation rate and inhibits apoptosis in colorectal cancer.^[51]^ This evidence supported a functional basis for the connection between these RNA editing sites and patients’ prognoses.

It is still not clear how these sites included in the model affect the survival of CESC patients. As reported, RNA editing can regulate gene expression levels by changing RNA sequences, which in turn can affect the translation and function of proteins.^[41]^ In the study, we observed significantly positive correlations between chr19:45896816 and PPP1R13L expression, chr22:36662801 and APOL1 expression, and chr14:55834429 and ATG14 expression. Since mRNA levels may not necessarily represent post-transcriptionally regulated protein levels for genes, the absence of significant correlation between the level of RNA editing at other 3 sites and host gene expression does not necessarily imply that these editing sites have no effect on host gene expression. Further analysis at the protein level is necessary to determine whether these sites may influence the prognosis of CESC patients by regulating host gene expression. Besides, ADAR, as the key regulator of RNA editing, is closely linked to RNA editing. Furthermore, dysregulations of ADAR expression and RNA editing are common in diverse human diseases.^[52,53]^ Our research also discovered a significant positive association between risk scores and ADAR expression levels.

Furthermore, we verified that this prognostic model can accurately forecast patients’ prognoses by survival analysis and ROC curve. Additionally, the significant and independent prognostic capacity of this risk model was further demonstrated after accounting for age, tumor stage, and tumor grade. To expand the clinical application and usability of RNA editing-based risk signatures, we combined risk score and clinical features to establish the nomogram with a high AUC value. Moreover, the calibration plot further revealed the good prognostic capability of this nomogram with high Harrell C-index as 0.844. By decision curves, the nomogram presented the better net benefit of clinical decisions. Overall, the nomogram showed good performance, accuracy, and stability in predicting OS in CESC.

Besides, we conducted an enrichment analysis to compare the differences in biological functions and pathways of DEGs among distinct risk subgroups. According to the findings of GO enrichment analysis, it could be observed that DEGs in different risk subgroups had significant changes in biological functions such as immune response, signal transduction, cell motility, and cell-cell interactions. These functional changes are intricately related to the proliferation and metastasis of tumor cell. KEGG analysis displayed that DEGs were connected with cytokine − cytokine receptor interaction, MAPK signaling pathway, IL − 17 signaling pathway, NF − kappa B signaling pathway, and TNF signaling pathway. These pathways all play an important role in immune response, inflammation, cell proliferation and tumor progression.^[54–58]^

GSEA was employed to further explore the biological function and pathway of DEGs among different risk subgroups. The results displayed that DEGs in the subgroup with high-risk were mainly enriched in cytokine-cytokine receptor interaction, ECM receptor interaction, focal adhesion, NOD-like receptor signaling pathway and regulation of actin cytoskeleton. Cytokine-cytokine receptor interaction is the important regulatory mechanism in physiological processes such as immunity and inflammation, and is strongly linked to the development and progression of cancer.^[54,59]^ Dysregulation of ECM receptor interaction affects tumor progression by promoting cell proliferation, differentiation, migration, and survival.^[60,61]^ Dynamic regulation of focal adhesion and regulation of actin cytoskeleton are key factors in cell migration, exerting significant effects on promoting the invasion of tumor cells.^[62]^ Also, the NOD-like receptor signaling pathway is associated with the immune system and regulates the development of cancer.^[63]^ Overall, we observed multiple biological functions and pathways correlated with immune response, cell proliferation, and tumor progression in the high-risk subgroup.

Currently, some limitations remain in our study. First, only data from CESC patients in the TCGA database were analyzed, lacking independent external data to enhance the credibility of this model. Secondly, because of the limited information about CESC patients provided by the TCGA database, the nomogram built in the study could not include relatively abundant clinical factors. Finally, rigorous experiments are required to further gain a deeper understanding of the intrinsic mechanism by which the identified RNA editing sites function in CESC.

## 5. Conclusions

We developed a risk model utilizing RNA editing and verified that it has good prognostic value for CESC patients. By integrating risk scores with clinical factors, the nomogram with good predictive performance was constructed. Through in-depth analysis, we found that RNA editing plays a crucial role in affecting tumor progression. It exerts its influence by modulating the proliferation, metastasis, and immune response of tumor cell within the tumor microenvironment.

## Author contributions

**Conceptualization:** Jing Lu, Zihan Zhu.

**Data curation:** Zihan Zhu.

**Formal analysis:** Zihan Zhu.

**Writing – original draft:** Zihan Zhu.

**Writing – review & editing:** Jing Lu.
